# The Role of Triacylglycerol in Plant Stress Response

**DOI:** 10.3390/plants9040472

**Published:** 2020-04-08

**Authors:** Junhao Lu, Yang Xu, Juli Wang, Stacy D. Singer, Guanqun Chen

**Affiliations:** 1 Department of Agricultural, Food and Nutritional Science, University of Alberta, Edmonton, T6G 2P5 Alberta, Canada; junhao3@ualberta.ca (J.L.); yxu9@ualberta.ca (Y.X.); juli1@ualberta.ca (J.W.); 2 Agriculture and Agri-Food Canada, Lethbridge Research and Development Centre, Lethbridge, T1J 4B1 Alberta, Canada

**Keywords:** triacylglycerol, stress tolerance, lipid remodeling, lipid droplets, storage lipid biosynthesis

## Abstract

Vegetable oil is mainly composed of triacylglycerol (TAG), a storage lipid that serves as a major commodity for food and industrial purposes, as well as an alternative biofuel source. While TAG is typically not produced at significant levels in vegetative tissues, emerging evidence suggests that its accumulation in such tissues may provide one mechanism by which plants cope with abiotic stress. Different types of abiotic stress induce lipid remodeling through the action of specific lipases, which results in various alterations in membrane lipid composition. This response induces the formation of toxic lipid intermediates that cause membrane damage or cell death. However, increased levels of TAG under stress conditions are believed to function, at least in part, as a means of sequestering these toxic lipid intermediates. Moreover, the lipid droplets (LDs) in which TAG is enclosed also function as a subcellular factory to provide binding sites and substrates for the biosynthesis of bioactive compounds that protect against insects and fungi. Though our knowledge concerning the role of TAG in stress tolerance is expanding, many gaps in our understanding of the mechanisms driving these processes are still evident. In this review, we highlight progress that has been made to decipher the role of TAG in plant stress response, and we discuss possible ways in which this information could be utilized to improve crops in the future.

## 1. Introduction

The growth of the global population, along with escalating per capita calorific consumption and need for plant-derived renewable resources, is leading to increased demand for crop products [1]. However, crop yields are negatively impacted by abiotic stresses such as heat, cold, drought, and salinity—the severity and frequency of which are increasing due to climate change [2,3]. The growing prevalence of abiotic challenges also increases the susceptibility of plants to biotic stresses such as pathogens and insects [4], which can further exacerbate crop yield losses. As sessile organisms, plants have evolved various molecular, metabolic, and physiological adaptations that aid in their ability to cope with a wide range of stresses [5], and while our knowledge surrounding these processes is expanding at a rapid pace, large gaps remain in our understanding. Due to the substantial negative effect abiotic and biotic stresses can have on yield, it is essential that stress response mechanisms are further elucidated. Such knowledge will almost certainly be requisite for the enhancement of stress tolerance, and, hence, the maintenance or increase of crop yield, under a future of climate change.

Triacylglycerol (TAG) is the major component of vegetative oils and is composed of a glycerol backbone bearing three esterified fatty acids. In plants, TAG is mainly stored as a high-energy storage compound within lipid droplets (LDs) in seeds or fruits [6]. Vegetative tissues such as leaves and stems, on the other hand, are typically regarded as non-lipid-storing tissues, at least under favorable environmental conditions, due to their limited contents of TAG. However, small amounts of TAG do exist in these tissues [6], where they have been suggested to play a role in the sequestration of toxic lipid intermediates following membrane breakdown and light-induced stomatal opening [7,8].

Over recent years, a growing number of studies have also linked vegetative TAG production to plant stress response, and the precise mechanisms behind such a function are in the process of being unraveled [9]. In this review, we examine our current understanding of TAG metabolism in vegetative tissues, as well as recent progress that has been made towards elucidating the role of vegetative TAG in stress tolerance. Furthermore, we also discuss the potential of modulating TAG metabolism as a means of increasing the resiliency of crop species to environmental challenges.

## 2. Triacylglycerol Metabolism in Vegetative Tissues

### 2.1. Triacylglycerol Biosynthesis

**Figure 1:**
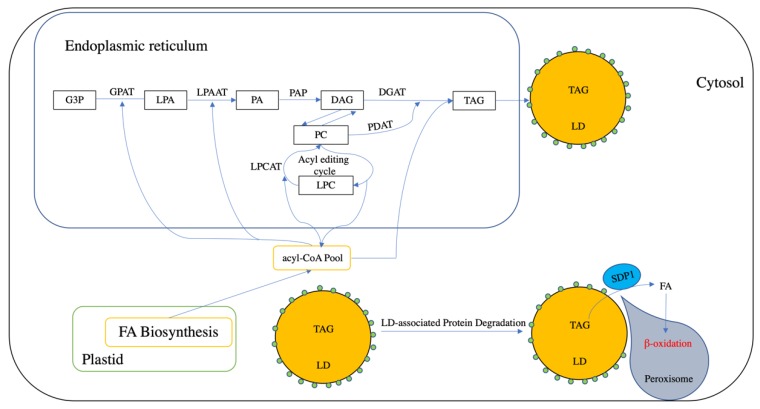
Brief Overview of triacylglycerol (TAG) biosynthesis, storage, and degradation in plants. Plastid provides a fatty acid (FA) pool, which can be transported into cytosol and activate into acyl-CoA. In the endoplasmic reticulum, G3P (glycerol 3-phosphate) can be acylated into lysophosphatidic acid (LPA) at the sn-1 position by using acyl-CoA as an acyl donor. Lyso-phosphatidic acid (LPA) can then be acylated with an acyl moiety from acyl-CoA pool and yield phosphatidic acid (PA) by acyl-CoA:lyso-phosphatidic acid acyltransferase (LPAAT). Phosphatidic acid phosphatase (PAP) is responsible for the dephosphorylation of PA into diacylglycerol (DAG). DAG can be directly acylated into TAG by diacylglycerol acyltransferase (DGAT). DAG also can be exchanged with phosphatidylcholines (PC). PC also can be generated through the acyl editing cycle, which involves the reacylation–acylation cycle. Additionally, acyl-CoA can be incorporated into PC by lysophosphatidylcholine acyltransferase (LPCAT). PC can be catalyzed by a phospholipid:diacylglycerol acyltransferase (PDAT) into TAG. Lipid droplets (LDs) is an organelle used for TAG storage, which is covered by a single layer of phospholipid- and LD-associated proteins (small green circles). The degradation of LD is initiated with the degradation of LD-associated proteins. Afterwards, peroxisome can interact with the LD and deliver a TAG lipase (sugar-dependent 1, SDP1) into the LD surface and hydrolysis TAG into FA and glycerol with the association of other lipases (not shown in this figure). FA is transported into peroxisome for β-oxidation.

In higher plants, TAG can either be synthesized and stored in the cytosol (within LDs) or in the chloroplast (within plastoglobules), termed cytosolic TAG and chloroplastic TAG, respectively [10,11]. The major route of cytosolic TAG biosynthesis requires de novo fatty acid (FA) biosynthesis and TAG assembly, both of which take place in distinct cellular compartments (Figure 1) [12]. Chloroplasts are the primary site of fatty acid biosynthesis, which begins with the conversion of pyruvate to acetyl-CoA. Fatty acyl chains 16 and 18 carbons in length are generated from acetyl-CoA and are then transported out of the plastid and activated into acyl-CoA for TAG biosynthesis [13].

The assembly of cytosolic TAG occurs on the endoplasmic reticulum (ER) via the Kennedy pathway using glycerol-3-phosphate (G3P) produced during glycolysis as the carbon backbone and acyl-CoA as the acyl donor (for reviews, refer to [6]). In this pathway, G3P is first acylated at the *sn*-1 position by *sn*-glycerol-3-phosphate acyltransferase (GPAT) to produce lysophosphatidic acid (LPA) [14,15], which is then acylated at the *sn*-2 position through the action of acyl-CoA:lysophosphatidic acid acyltransferase (LPAAT) to yield phosphatidic acid (PA) [16]. Mg^2+^-dependent phosphatidic acid phosphatases (PAP) then converts the PA into *sn*-1,2-diacylglycerol (DAG) [17]. The final step is catalyzed by diacylglycerol acyltransferase (DGAT), which acylates the *sn*-3 position of DAG to yield TAG [18].

De novo synthesized 18:1-DAG can also be channeled into 18:1-phosphatidylcholine (PC) through the catalytic action of Cytidine 5’-diphosphocholine-choline:diacylglycerol choline phosphotransferase (CPT) [19] and phosphatidylcholine:diacylglycerol choline phosphotransferase (PDCT) [20]. PC is regarded as the major site for the modification of FAs, where 18:1-PC is converted into polyunsaturated PC-modified FA (PC-mFA) via the acyl-editing cycle, which involves de-acylation and re-acylation but does not generate new PC [21]. In addition, plastid 18:1 acyl-CoA can also be directly incorporated into PC-mFA through the activity of lysophosphatidylcholine acyltransferase (LPCAT) [17] (for review, refer to [22]). Phospholipid:diacylglycerol acyltransferase (PDAT) is then able to use PC-mFA as an acyl donor to catalyze the acylation of DAG into TAG, producing lysoPC as a by-product. This route is termed the acyl-CoA-independent pathway, which differs from the Kennedy pathway [23]. However, the relative contribution of these two routes in terms of TAG biosynthesis is not equal in vegetative tissues. For example, in *Arabidopsis*, PDAT1 is responsible for the majority of TAG biosynthesis in growing leaves, whereas DGAT1 plays a more important role in senescing leaves [24,25,26].

In vegetative tissues, a large proportion of FAs can also be utilized for the production of plastid membrane lipids, especially in leaves (for review, refer to [27]). Specifically, GPAT1 [28] and LPAAT1 [29] are responsible for plastid-localized reactions that produce PA, which is then converted into DAG through the action of plastid-localized lipid phosphate phosphatase (LPP) in *Arabidopsis* [30]. Plastidic DAG can then be incorporated into glycerolipids, such as monogalactosyldiacylglycerol (MGDG), digalactosyldiacylglycerol (DGDG), and sulfolipidsulfoquinovosyldiacylglycerol (SQDG) [27]. The thylakoid localized phytyl ester synthase 1 (PES1) and PES2 then contribute to chloroplastic TAG biosynthesis by converting DAG into TAG [31]. However, the precise pathway driving the acylation of DAG to produce TAG in cholroplastids has still not been fully elucidated, and it is likely that other, as of yet unidentified, acyltransferases are also involved [27].

### 2.2. Triacylglycerol Storage

In the cytosol, increasing amounts of nascent TAG between two membrane leaflets of the ER can induce the budding of LDs, which are subcellular organelles composed of a neutral lipid core (e.g., TAG) covered with a monolayer of phospholipid (PL) and surface proteins [10]. Until recently, LDs were viewed simply as neutral lipid storage organelles; however, they are now believed to provide dynamic functions in various physiological processes (for review, refer to [10,11]). The composition of the LD proteome often varies among plant tissues, development stages, and growth conditions [32]. For example, the proteome composition of leaf and seed LDs differs, and in *Arabidopsis*, leaf LD proteomes are mainly composed of small rubber particle protein (SRP), caleosin, and dioxygenase [33]. Intriguingly, such LD-associated proteins have been suggested to be involved in stress response [10], and their precise functions are discussed in Section 5.

Plastidic TAG, on the other hand, is stored in plastoglobules, which can be found in curved regions of the thylakoid membrane system [34], and like LDs have been recognized to function in a variety of physiological, metabolic, and developmental processes. The structure of plastoglobules is similar to that of LDs; however, the composition of the plastoglobule core differs, incorporating TAG, fatty acid phytyl ester (FAPE), or carotenoids. Unlike cytosolic LDs, the most abundant proteins associated with plastoglobules include the FIBRILLIN (FBN) and ACTIVITY OF BC1 COMPLEX KINASE (ABC1K) family proteins (for review, refer to [35]). Moreover, PES1 and PES2, which contribute to plastid TAG biosynthesis, are also found in the plastoglobule proteome [31].

## 3. Induction of TAG Accumulation under Stress Conditions

Though TAG typically does not accumulate to substantial levels in vegetative tissues under non-limiting growth conditions, various stress conditions such as drought [36], high or low temperature, and nutrient starvation [37] can induce its production, especially in leaves. This has been attributed to the up-regulation of several key genes involved in TAG biosynthesis. Abscisic acid (ABA) is a plant hormone that accumulates under stress and functions as a signaling molecule to regulate plant development and metabolic pathways under a wide range of abiotic stress conditions [38]. ABA is also known to be involved in lipid accumulation in developing seeds, and both developing embryos and cell cultures treated with ABA produce elevated levels of TAG, as well as increased proportions of polyunsaturated and long-chain fatty acids (e.g., [39,40]). In vegetative tissues, the transcription factor ABSCISIC ACID INSENSITVE 4 (ABI4), which acts as a key component in the ABA signaling pathway and is up-regulated under stress conditions, has been found to bind the *DGAT1* promoter to increase its expression in *Arabidopsis* [41]. Similarly, *ABI5* has also been found to synergistically up-regulate the expression of *DGAT1* under nitrogen deprivation in *Arabidopsis* [42], while the ABA-inducible MYB96 transcription factor induces the expression of *DGAT1* (indirectly) and *PDAT* (directly) in both vegetative tissues and seeds [43]. Taken together, these findings suggest a tight linkage between stress-induced ABA accumulation and TAG biosynthesis in vegetative tissues.

The expression of other genes involved in TAG biosynthesis has also been shown to be increased under stress conditions. For example, *Arabidopsis LPAAT4* and *LPAAT5*, which are responsible for providing the DAG substrate for TAG biosynthesis in the Kennedy pathway, have been found to be up-regulated under nitrogen deprivation [37]. This transcriptional modulation has been suggested to result in an increased supply of DAG for TAG production in vegetative tissues [37].

## 4. Role of TAG in Stress Response by Sequestering Toxic Lipid Intermediates 

Under various stress conditions, plant cells undergo substantial alterations to both plastidic and extraplastidic membrane lipid composition via lipid remodeling, which aids in the maintenance of membrane fluidity, stability, and integrity [44]. Environmental stress can induce the degradation of MGDG and chlorophyll in chloroplasts [27], which results in the accumulation of toxic lipid intermediates (including DAG, free fatty acids (FFAs), and phytyl) that can damage plant cells. In many cases, TAG appears to act as a transit pool to sequester some of these toxic intermediates, thus preventing cellular damage under stress conditions. In *Arabidopsis*, the plastoglobule-localized acyltransferases PES1 and PES2 have been found to provide a detoxifying role by catalyzing the conversion of DAG into TAG and using acyl-CoA, galactolipids, and acyl carrier proteins as acyl donors. Meanwhile, PES1 and PES2 can also convert FFA and phytyl into FAPE as a means of detoxification [31]. While the expression levels of *PES1* and *PES2* have been found to increase under nitrogen starvation and senescence [31], it remains to be determined whether they are also involved in the response to other types of stress.

**Figure 2:**
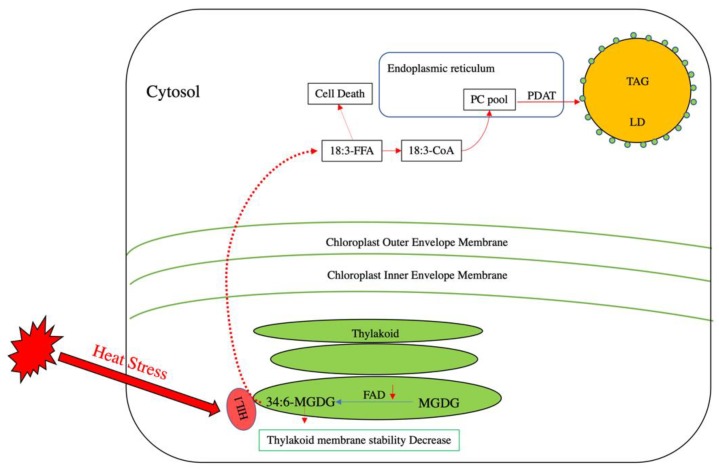
Lipid remodeling in *Arabidopsis* under heat stress. In thylakoid membranes, monogalactosyldiacylglycerol (MGDG) and fatty acid desaturase (FAD) introduce double bonds into MGDG to yield 34:6-MGDG. Under heat stress, polyunsaturated fatty acid-enriched MGDG can decrease thylakoid membrane stability. The activity of some FADs decreases to reduce the production of 34:6-MGDG. Concomitantly, a putative thylakoid-localized lipase termed heat-induced lipase 1 (HIL 1) is activated under heat stress to release 18:3 free fatty acid (18:3-FFA) from 34:6-MGDG. The release of 18:3 FFA can induce cell death. To prevent such damage, 18:3-FFA can be activated into 18:3-CoA and is believed to be incorporated into the PC pool. PDAT then utilizes the derived PC to produce TAG to sequester the 18:3-FFA and reduce heat-induced damage.

During heat stress, the degree of membrane lipid acyl chain unsaturation plays a vital role in maintaining membrane function. Under such conditions, plants need to decrease the ratio of unsaturated to saturated fatty acids in their membrane lipid composition to decrease membrane fluidity and prevent hyperfluidity damage [44]. While plants tend to reduce the activity of various fatty acid desaturases (FADs) under high temperatures as a means of reducing desaturation of membrane lipids [45,46,47], another way in which chloroplast membrane stability can be achieved is through the removal of polyunsaturated acyl groups from MGDG. In *Arabidopsis*, a chloroplast-localized lipase, *HEAT INDUCIBLE LIPASE1* (*HIL1*), releases free α-linolenic from 18:3-enriched MGDG to decrease the content of polyunsaturated acyl groups under heat stress [48]. However, since FFAs at high levels are toxic to cells and their over-accumulation can induce cell death [48,49,50], 18:3 is transported from the chloroplast stroma onto the ER membrane, where it is activated into 18:3-CoA. Based on lipidomic analyses, it appears that this 18:3-CoA is mainly channeled into PC [50], which is used as an acyl donor by PDAT to sequester the 18:3 into TAG (Figure 2) [48,50]. Indeed, PDAT has been shown to be involved in heat stress tolerance by converting FFAs derived from membrane degradation into TAG via PC [48,50]. Therefore, the accumulation of TAG under heat stress likely plays a major role in adaptation by sequestering the toxic FFA by-products of membrane lipid remodeling.

**Figure 3:**
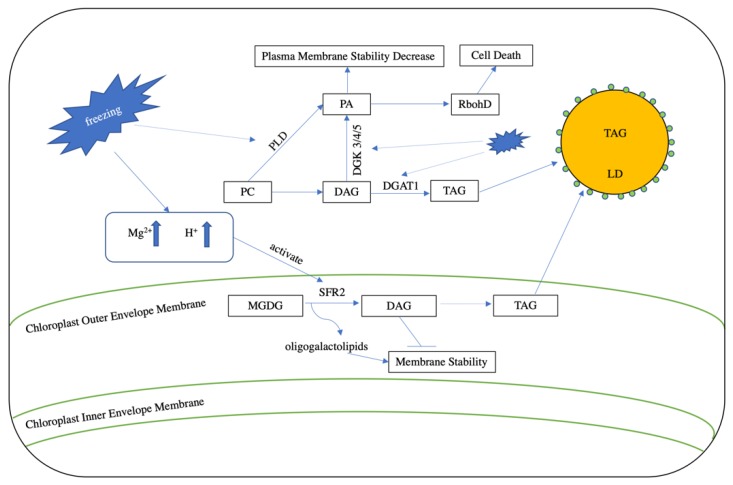
Lipid turnover under freezing stress. During freezing stress, cellular dehydration increases the concentration of Mg^2+^ and cellular acidity. The induced cellular leakage then transports Mg^2+^ and protons into the chloroplast outer envelope membrane and activates the function of SENSITIVE TO FREEZING2 (SFR2). SFR2 converts MGDG into oligogalactolipids and DAG. Oligogalactolipids enhance membrane stability, whereas DAG reduces stability. DGAT1 can sequester DAG into TAG to decrease membrane damage. In addition, freezing stress also elevates PA levels through the action of phospholipase D (PLD) and the DAG kinase 3/4/5 (DGK 3/4/5). This increase in PA decreases plasma membrane stability and induces the formation of NADPH oxidase (RbohD), which in turn leads to the production of reactive oxygen species (ROS) and cell death.

As is the case with excessive levels of heat, cold stress can also severely restrict plant growth and decrease crop yields. Under freezing stress, plants accumulate DAG, largely through the activity of *SENSITIVE TO FREEZING2* (*SFR2*), which encodes a galactolipid:galactolipid galactosyltransferase (GGGT) that is localized to the outer chloroplast envelope [51]. Freezing stress induces the formation of apoplastic ice and cellular dehydration. These both lead to membrane leakage, which results in the release of Mg^2+^ and cellular acidification [52], as well as the consequential post-translational activation of SFR2. Once activated, this enzyme transfers galactosyl groups from MGDG into galactolipid acceptors, leading to increased production of oligogalactolipids and DAG [52]. While oligogalactolipids can serve to enhance the stability of the chloroplast membrane [53], DAG is released into the cytosol during membrane shrinkage (which is caused by freezing) [53,54], becoming available for conversion to PA via the catalytic action of the DAG kinase (DGK; Figure 3). Though the freezing-induced accumulation of PA occurs mainly through the action of DGK, the hydrolysis of membrane phospholipids by phospholipase D also contributes under these conditions [55].

The resulting PA directly binds to the PA-binding motif in the N-terminal region of NADPH oxidase (RbohD), stimulating its activity and thus increasing the production of the reactive oxygen species (ROS) superoxide [56]. While small amounts of ROS play important roles in stress response by acting as signal transduction molecules [57], they are harmful when present above a certain threshold, leading to the oxidation of membrane lipids, as well as protein, chlorophyll, and nucleic acid damage (Figure 3; [58]) [56]. In addition, PA can also induce the formation of an unstable hexagonal II (H_II_)-type lipid phase with DAG or MGDG [53,59], which damages the cell membrane during freezing-induced dehydration [52].

Recently, growing evidence has suggested that TAG plays an important role in the ability of plants to withstand freezing stress by modulating the relative conversion of excess DAG into TAG instead of PA via the catalytic actions of DGAT and DGK. For example, the expression of *Arabidopsis DGAT1* is up-regulated under low temperature conditions, and *dgat1* mutants display increased sensitivity to freezing stress compared to wild-type plants. In line with this, a comparative genomics study between freezing sensitive and tolerant *Boechera stricta* lines indicated that the up-regulation of *DGAT1* might provide a common mechanism for conferring freezing stress tolerance [60], feasibly because the increased conversion of DAG to TAG would be associated with a decrease in DAG-to-PA reactions. Correspondingly, the *Arabidopsis dgk2*, *dgk3*, and *dgk5* triple mutant, which exhibits a reduction in DAG-to-PA conversion, displays improved freezing tolerance compared to controls [53].

In a similar manner to freezing stress, drought and salinity conditions also cause cellular dehydration, which induces the accumulation of Mg^2+^ and lowers cytosolic pH. In line with this, the *SFR2* homolog (*SlSFR2*) from a cold-sensitive tomato (*Solanum lycopersicum*) has also been found to function in salt and drought response via a similar post-translational activation mechanism to that used during exposure to low temperature stress [61]. However, this broad-spectrum effect has not always been found to be the case, since *Arabidopsis SFR2* does not appear to contribute to resiliency against salinity or drought stress [61]. One possible explanation for this finding is that *SFR2* orthologs in different species may encode proteins with differing sensitivities to Mg^2+^. Therefore, the identification and over-expression of *SFR2* orthologs with high sensitivities to Mg^2+^ [61], along with *DGAT1* co-expression to promote the sequestration of excess DAG into TAG rather than PA could be a promising way to engineer crops with an improved tolerance to a wide variety of abiotic stresses in the future.

## 5. Role of Cytosolic Lipid Droplets in Stress Response

While only small numbers of LDs are present in vegetative tissues (e.g., leaves) under non-limiting growth conditions [33], they have been found to accumulate in vegetative tissues during senescence [62], as well as under stresses such as drought [63] and fungal infection [64]. In *Arabidopsis*, three isoforms of small rubber particle proteins (SRPs) are primarily responsible for maintaining LD stability in leaves [63], and the over-expression or suppression of *SRP*s has been found to result in an increased or decreased number of LDs, respectively, in leaf tissues. Furthermore, the disruption of *SRP*s has also been found to reduce tolerance to drought and high temperatures [63]. Since LDs provide a scaffold for the binding of enzymes that function in stress tolerance and the provision of substrates for the biosynthesis of bioactive compounds, it is possible that the reduction in LDs observed following the disruption of *SRP*s simply leads to the lack of an adequate binding surface for these LD-associated proteins [65]. Therefore, it stands to reason that the over-expression of *SRP*s and the corresponding increase in LD number in leaves could enhance the binding of LD-associated proteins and potentially ameliorate stress tolerance. While this has yet to be assessed, its further study is certainly warranted because it may provide another possible approach for improving resiliency to various types of stress.

Caleosin, which can sense alterations in Ca^2+^ concentrations that typically occur during stress-related signalling [65,66,67], is an abundant LD-associated protein. RESPONSIVE TO DEHYDRATION20 (RD20; also known as Caleosin3) is a specific isoform of caleosin found in *Arabidopsis* leaf LDs, the disruption of which leads to increased stomatal apertures and transpiration rates, thus decreasing drought tolerance [67]. Similarly, *RD20* loss-of-function mutants are also more sensitive to salinity stress [67]. However, the over-expression of *RD20* does not confer increased drought resistance [67], which may stem from the fact that the function of RD20 depends on its appropriate localization to leaf LDs by SRPs in *Arabidopsis* [63]. Thus, it is possible that the co-expression of *SRP*s along with *RD20* might be required to elicit improvements in stress tolerance. Since different caleosins are also active during pollen germination, their possible function in stress response in this context may also be worth further investigation [68,69].

In addition to their Ca^2+^ sensing ability, caleosins also possess peroxygenase (PXG) activity, which is responsible for the epoxidation and hydroxylation of polyunsaturated FAs [70]. This PXG activity also allows caleosins to be involved in the biosynthesis of oxygenated lipids (oxylipins), which are implicated in defense against biotic stresses such as pathogens and herbivores [71]. In *Arabidopsis*, α-dioxygenase (α-DOX) co-localizes on the surface of leaf LDs with RD20, and it catalyzes the production of an antifungal compound termed 2-hydroxy-octacdecatrienoic acid (2-HOT) that defends against the pathogenic fungus, *Colletotrichum higginsianum* [72]. RD20 also utilizes 13-hydro-peroxyoctadecatrienoic acid (13-HPOT) as a substrate for the production of phytoalexins, such as 13-hydroxyoctadecatrienoic acid (13-HOT) and 15,16-epoxy-13-HOT [73], which protect against fungal and oomycete infection [74]. Among all of the above reactions, α-linolenic acid is essential as a common substrate of 2-HPOT and 13-HPOT. While this FA was originally believed to be produced by an LD-localized lipase (e.g., SUGAR DEPENDENT1 (SDP1)), its origin is still not clear, and it has been suggested that it may instead be derived from stress-induced membrane lipid remodeling [9].

Lipoxygenases (LOX) play an important role in polyunsaturated fatty acid oxidation, the products of which act as substrates for downstream reactions that yield biologically active oxylipins [74] and are known to be involved in various developmental processes, including stress tolerance (e.g., [75]). Putative LD-localized 13-lipoxygenases (13-LOXs), for example, utilize 18:3 to produce 13-hydroperoxyoctadecatrienoic acid (13-HPOT), which is a precursor for RD20-mediated 13-HOT biosynthesis [76]. In addition, 13-HPOT can also be used as a substrate to produce a variety of other oxylipins with protective roles against stress through the action of enzymes such as allene oxide synthase (AOS), allene oxide cyclase (AOC), and hydroperoxide lyase (HPL) (see [77,78] for reviews). In line with this, the overexpression of a gene encoding 13-LOX in barley (*Hordeum vulgare L*) has been found to lead to a decrease in the fecundity of green peach aphid (*Myzus persicae Sulzer*) [79] when feeding on the resulting lines, which hints at the potential of manipulating LOXs for crop improvement in the future.

## 6. Closing Comments

While abiotic and biotic stresses can lead to significant reductions in both seed and fruit oil production [80,81], increases in TAG in vegetative tissues are often observed. To date, the roles of TAG in stress response have been partially elucidated by using a combination of omics and molecular approaches. One mechanism by which plants cope with stress is through alterations in membrane lipid composition via lipase-mediated lipid remodeling. The lipid by-products of this process are toxic, but increased levels of TAG resulting from the up-regulation of *DGAT* or *PDAT* allows for detoxification through their sequestration. Moreover, LDs also provide substrates and binding sites for LD-associated proteins, which synthesize bioactive compounds that can increase tolerance to biotic stresses. The establishment of a relationship between the transcriptome and lipidome of *Arabidopsis* under different stress conditions has provided a suite of candidate genes that may be involved in lipid remodeling to confer stress tolerance [82,83]. While these studies have certainly provided vital information, this approach will fail to identify those candidate genes, such as SFR2, with transcript levels that do not vary under stress and are instead regulated at the protein level. 

Our increasing understanding of TAG metabolism and expansion in molecular breeding tools has made it possible to manipulate plant oil accumulation, which might provide a plausible means of modulating plant stress tolerance. However, it has yet to be determined whether increasing oil content in storage or vegetative tissues can contribute to stress tolerance in crop species. While existing evidence certainly points to the feasibility of such an approach, a high leaf-oil transgenic *Arabidopsis* line was found to become more susceptible to heat stress and cabbage looper (*Trichoplusia ni*) [84]. Therefore, it is evident that the systemic evaluation of the performance of such crops under different stresses and cropping systems, as well as the fatty acid compositions of their vegetative tissues, will be necessary to gain full insight into the matter. Furthermore, the co-manipulation of other stress-related genes such as those encoding LD-associated proteins might also be required for the development of high-oil, stress-tolerant cultivars in the future.
